# Biogenic silver nanoparticle coupled MnO_2_/MXene hybrid for enhanced electrochemical performance in supercapacitors

**DOI:** 10.1039/d6ra06185k

**Published:** 2026-10-20

**Authors:** Hilal Özdemir, Fatma Kiliç Dokan, Senem Sanduvaç, Ertugrul Sahmetlioglu

**Affiliations:** a Giresun University, Faculty of Arts and Sciences, Block B, Department of Physics Güre Campus Giresun 28200 Türkiye; b Department of Chemistry and Chemical Processing Technologies, Mustafa Çıkrıkcıoglu Vocational School, Kayseri University Kayseri Türkiye fatmakilic@kayseri.edu.tr; c Bünyan Vocational School, Kayseri University Kayseri 38039 Turkey; d Department of Basic Sciences of Engineering, Kayseri University Kayseri 38039 Türkiye sahmetlioglu@kayseri.edu.tr

## Abstract

The development of high-performance, sustainable electrodes remains a key challenge for next-generation supercapacitors. A ternary hybrid of MnO_2_, MXene, and biogenic silver nanoparticles (AgNPs) was designed as an advanced electrode material. AgNPs were synthesized *via* a green, cost-effective route using *Camellia* stamen extract, providing environmental compatibility and functional surface chemistry. The MnO_2_/MXene framework combines the high theoretical capacitance of MnO_2_ with the excellent conductivity of MXene. Incorporation of AgNPs enhances electrical pathways and accelerates charge transfer at the electrode–electrolyte interface. Structural analyses confirm uniform AgNP distribution, increasing active surface area and electrochemical accessibility. Electrochemical tests show that the AgNP-integrated MnO_2_/MXene electrode delivers superior capacitance, rate capability, and cycling stability compared to binary analogues. The enhanced performance arises from the synergistic interaction of MnO_2_, MXene, and AgNPs, which supports both surface-controlled and diffusion-assisted charge storage processes. This approach highlights the potential of green-synthesized nanomaterials for efficient hybrid electrode design.

## Abstract 2

MnO_2_/MXene/AgNP ternary electrodes were built for supercapacitors: MXene boosts conductivity, green-synthesized AgNPs (*Camellia* extract) speed charge transfer. Synergy yields higher capacitance, rate capability and stability than binary electrodes. Image created with Google Gemini.

## Graphical abstract

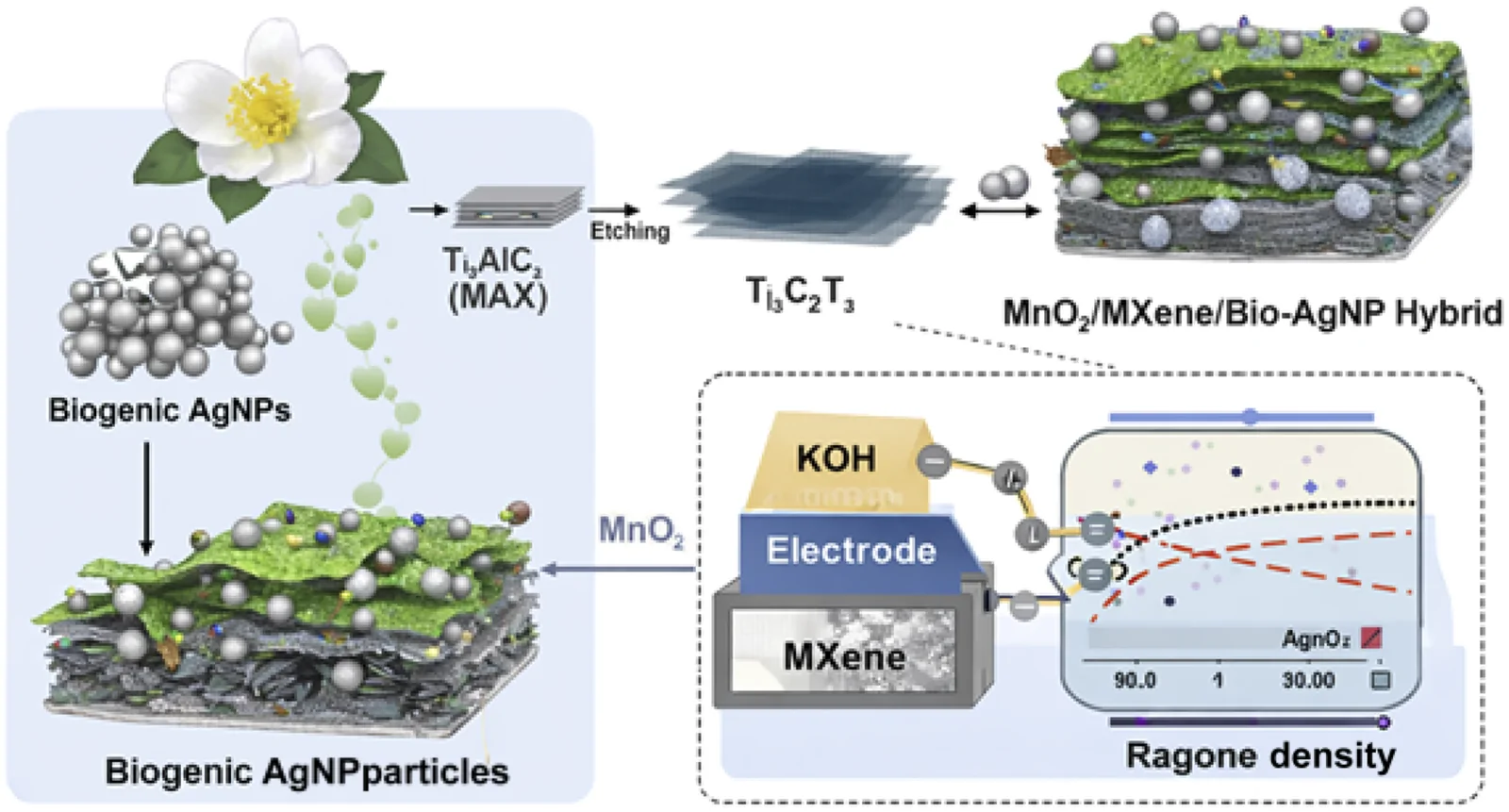

## 1 Introduction

The increasing demand for efficient and sustainable energy storage systems has accelerated the development of advanced electrode materials with high power density, rapid charge–discharge capability, and long-term cycling stability.^1–5^ Among various energy storage technologies, supercapacitors have attracted considerable attention due to their superior power performance and operational reliability. However, their relatively low energy density compared to battery systems remains a major limitation, largely governed by the intrinsic properties of electrode materials.^6–8^

Transition metal oxides, particularly manganese dioxide (MnO_2_), have been widely investigated as promising pseudocapacitive materials owing to their high theoretical capacitance, natural abundance, and environmental compatibility. Despite these advantages, the practical performance of MnO_2_ is often hindered by its low electrical conductivity and limited ion diffusion kinetics, which restrict charge transport during high-rate operation. To overcome these limitations, coupling MnO_2_ with conductive carbon-based materials has emerged as an effective strategy to enhance electrochemical performance.^9–13^

Among these materials, two-dimensional transition metal carbides and nitrides, known as MXenes, have gained increasing attention due to their high electrical conductivity, hydrophilic surface, and tunable surface terminations. MXene-based structures provide a conductive network that facilitates rapid electron transport and improves electrolyte accessibility. When combined with MnO_2_, MXene not only enhances electrical conductivity but also prevents structural aggregation, leading to improved electrochemical utilization of active sites.^14–17^

In addition to conductive supports, the incorporation of metallic nanoparticles has been shown to further improve charge transfer kinetics and interfacial reactions.^18^ Silver nanoparticles (AgNPs), in particular, exhibit excellent electrical conductivity and surface activity, making them attractive for hybrid electrode design. Conventional synthesis routes for AgNPs, however, often involve toxic reagents and complex processing steps. In contrast, green synthesis approaches using plant-derived extracts offer a simple, low-cost, and environmentally benign alternative. The use of *Camellia* stamen extract provides natural reducing and stabilizing agents, enabling the formation of biogenic AgNPs with functional surface characteristics.^19–22^

Integrating biogenic AgNPs into MnO_2_/MXene systems is expected to introduce additional conductive pathways and improve interfacial charge transfer. Furthermore, the presence of Ag nanoparticles can promote more efficient utilization of redox-active sites and facilitate faster ion–electron coupling within the electrode structure.^23^ Although MnO_2_/MXene/Ag-based hybrid electrodes have previously been investigated, the use of plant-mediated Ag nanoparticles and the systematic assessment of their contribution to the interfacial and electrochemical behavior of MnO_2_/MXene electrodes remain comparatively less explored. In this context, the present study focuses on the role of biogenic AgNPs rather than claiming novelty for the ternary electrode architecture itself. Although the absolute specific capacitance achieved in this study is lower than some previously reported chemically-synthesized Ag–MnO_2_/MXene systems,^57^ the present work demonstrates that a plant-mediated AgNP synthesis route can achieve comparable performance to conventional methods while offering a more sustainable synthesis pathway, alongside new mechanistic insight into the role of Ag in the charge-storage mechanism,^57,58^ the present study instead employs a plant-mediated (green) AgNP synthesis route and provides a systematic kinetic analysis (*via* power-law *b*-value and Dunn's-method decomposition) of how biogenic AgNP incorporation influences the capacitive/diffusive charge-storage balance of the MnO_2_/MXene framework—an aspect not addressed in these prior reports.^24^

In this context, the present study aims to design and fabricate a high-performance ternary hybrid electrode based on MnO_2_, MXene, and *Camellia* stamen-mediated (plant-mediated) biogenic Ag nanoparticles. The primary objective is to establish a structure that simultaneously improves electrical conductivity, accelerates charge transfer, and enhances the utilization of electroactive sites.^25–29^ Particular attention is given to understanding how the incorporation of biogenic AgNPs influences the charge storage mechanism, including the balance between surface-controlled and diffusion-controlled processes. The contribution of each component within the hybrid architecture is systematically evaluated to clarify their synergistic roles in governing electrochemical behavior.^30^

The significance of this work lies in incorporating a plant-mediated (green) AgNP synthesis step into an advanced hybrid material design, demonstrating how a sustainable nanoparticle synthesis route can be integrated into high-performance electrode architectures. By introducing plant-derived Ag nanoparticles into a MnO_2_/MXene framework, this study not only improves electrochemical performance but also provides insight into the design of multifunctional electrode systems. The findings are expected to contribute to the development of next-generation energy storage materials with improved efficiency, stability, and sustainability.

## 2 Materials and methods

### 2.1 Chemicals and materials

Titanium aluminum carbide (Ti_3_AlC_2_ MAX phase, ≥98%), lithium fluoride (LiF), hydrochloric acid (HCl, 37%), potassium permanganate (KMnO_4_), manganese sulfate monohydrate (MnSO_4_·H_2_O), silver nitrate (AgNO_3_), and potassium hydroxide (KOH) were obtained from commercial suppliers and used without further purification. Fresh *Camellia japonica* flowers were collected locally, and the stamens were separated and used as a natural reducing agent. Deionized (DI) water (18.2 MΩ cm) was used throughout all experiments.

### 2.2 Preparation of Ti_3_C_2_T_*x*_ MXene

Few-layer Ti_3_C_2_T_*x*_ MXene was synthesized *via* a modified minimally intensive layer delamination (MILD) method. Briefly, 1 g of LiF was dissolved in 20 mL of 9 M HCl under continuous stirring, followed by the slow addition of 1 g Ti_3_AlC_2_ powder. The etching reaction was maintained at 35 °C for 24 h to selectively remove the Al layers.^31^

The resulting suspension was washed repeatedly with DI water *via* centrifugation (3500 rpm) until the pH reached ∼6. The sediment was then sonicated under Ar atmosphere for 1 h to achieve delamination. The supernatant containing few-layer MXene sheets was collected and stored under inert conditions to prevent oxidation.

### 2.3 Green synthesis of biogenic Ag nanoparticles (Bio-AgNPs)

#### 2.3.1 Materials

Silver nitrate (AgNO_3_ >99% purity) was obtained from (KİMYALAB) and used without further purification. Deionized water was used for the preparation of all aqueous solutions. *Camellia japonica* flowers were freshly collected, and the stamens (anthers) were manually separated for the green synthesis process.

#### 2.3.2 Green extraction of *Camellia* stamens

The separated stamens were precisely weighed (2.027 g) and added to 100 mL of deionized water. The mixture was heated at 60 °C and stirred continuously for 30 minutes to facilitate the extraction of bioactive reducing and stabilizing agents. The resulting extract was allowed to cool to the room temperature and then filtered through Whatman no. 1 filter paper to remove any botanical residues. The clear extract was stored at 4 °C for further use.^32,33^

#### 2.3.3 Synthesis and purification of silver nanoparticles (AgNPs)

In a typical biogenic synthesis procedure, 10 mL of the *Camellia* stamen extract was mixed with 10 mL of 1 mM AgNO_3_ solution. The reaction was maintained under constant magnetic stirring for 60 minutes at 60 °C. The formation of AgNPs was visually confirmed by the transition of the solution color from pale yellow to a deep reddish-brown.^34^ A preliminary indication of AgNP formation was observed as a visual transition of the solution color from pale yellow to a deep reddish-brown, consistent with reports of biogenic Ag nanoparticle synthesis.^34^ The presence of metallic Ag in the final composite was subsequently confirmed by XRD, EDX, and STEM analyses (Section 3); however, independent verification of the nanoparticle formation step (*e.g.*, *via* UV-vis SPR analysis, DLS, or TEM sizing) was not performed in this study and is acknowledged as a limitation.

To ensure high purity for the subsequent hybrid electrode preparation, the synthesized AgNPs were collected *via* high-speed centrifugation at 15 000 rpm for 20 minutes. The obtained pellet was re-dispersed in deionized water and washed twice by further centrifugation at 15 000 rpm for 10 minutes to remove any unreacted precursors and excess plant metabolites.^35^

### 2.4 *In situ* growth of MnO_2_ on MXene sheets

MnO_2_ was directly grown on MXene sheets *via* a redox-assisted deposition method. Typically, 50 mL of MXene dispersion (2 mg mL^−1^) was mixed with 0.05 M KMnO_4_ solution under stirring. The reaction was carried out at 70 °C for 6 h.

During this process, MnO_4_^−^ ions were reduced on the MXene surface, leading to the formation of ultrathin MnO_2_ nanosheets anchored onto conductive MXene layers. The obtained MnO_2_/MXene composite was washed and vacuum dried at 60 °C.^36^

### 2.5 Construction of Ag–MnO_2_/MXene ternary hybrid

The ternary hybrid electrode material was synthesized *via* a two-step interfacial assembly strategy;^37,38^

Electrostatic adsorption: MnO_2_/MXene composite was dispersed in DI water and mixed with Bio-AgNPs under ultrasonication for 30 min to ensure homogeneous distribution.

*In situ* reduction-assisted anchoring (critical novelty): a small amount of *Camellia* extract was reintroduced to promote surface functional group interaction, enabling strong anchoring of AgNPs onto MnO_2_/MXene interfaces.

The mixture was stirred for 4 h, collected *via* centrifugation, washed, and dried. Different AgNP loadings (5, 10, and 15 wt%) were initially prepared and screened to identify a suitable loading for further characterization. Based on this preliminary comparison, the 10 wt% AgNP loading was selected for detailed structural and electrochemical characterization, as it provided the most favorable balance between Ag dispersion, structural integrity, and electrochemical response. All results presented in Section 3 correspond to this 10 wt% Ag–MnO_2_/MXenecomposite (Fig. 1).

**Fig. 1:**
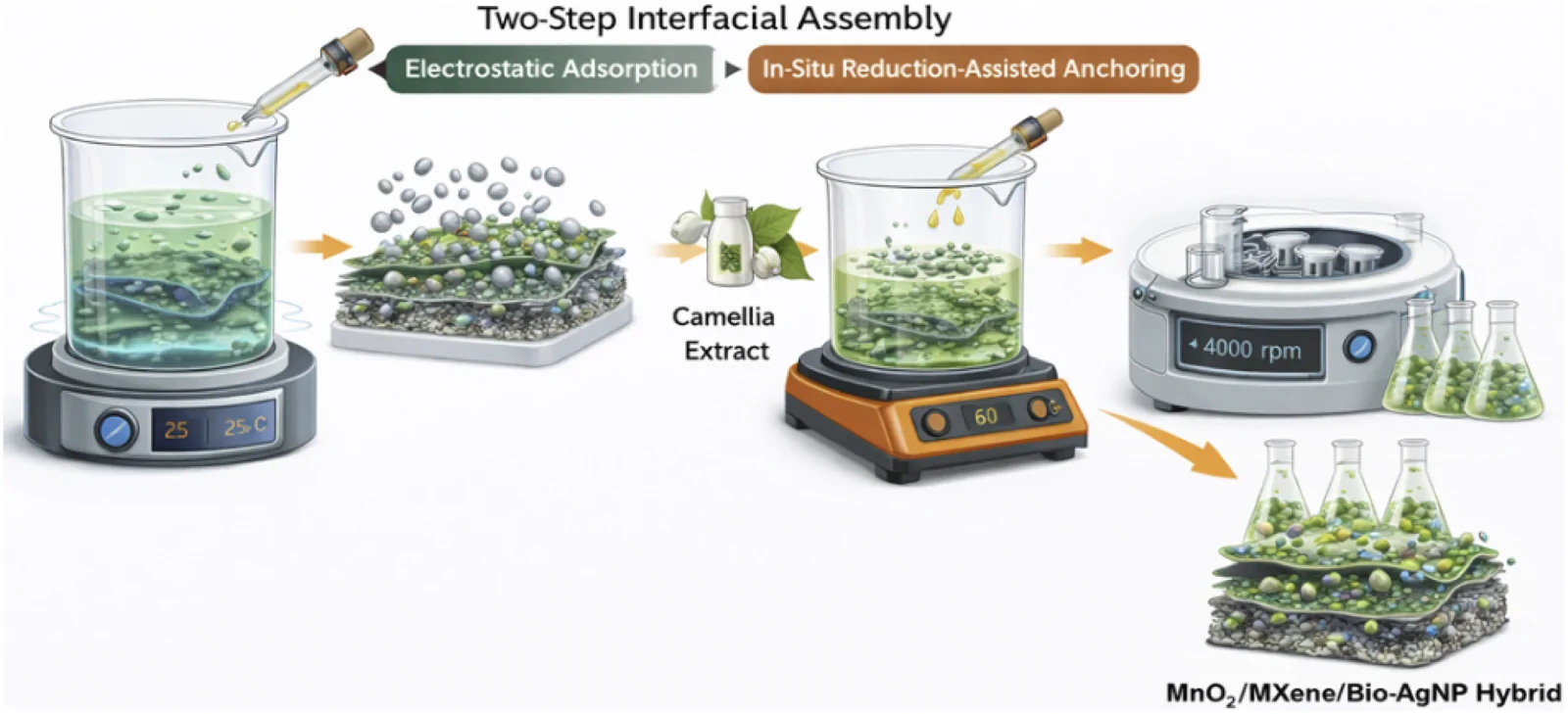
Two-step interfacial assembly of Ag–MnO_2_/MXene hybrid.

### 2.6 Material characterization

The morphology and microstructural features of the synthesized Ag–MnO_2_/MXene hybrid materials and corresponding electrodes were investigated using field-emission scanning electron microscopy (FE-SEM, Gemini 550, Zeiss). High-resolution imaging was employed to evaluate the surface architecture and the interfacial integration between MnO_2_ nanosheets, MXene layers, and biogenic Ag nanoparticles.

The crystalline structure and phase composition of the samples were analyzed by X-ray diffraction (XRD, Bruker AXS D8 Advance) using Cu-Kα radiation (*λ* = 0.15406 nm) over a 2*θ* range of 5–80°. The diffraction patterns were used to confirm the successful formation of MnO_2_, the preservation of MXene structure, and the incorporation of Ag nanoparticles.

Elemental composition and spatial distribution of Mn, Ti, O, C, and Ag within the hybrid structure were determined by energy-dispersive X-ray spectroscopy (EDX) coupled with FE-SEM (Zeiss). Elemental mapping analysis was conducted to verify the homogeneous dispersion of biogenic Ag nanoparticles and their interaction with the MnO_2_/MXene framework.

The specific surface area, pore volume, and pore size distribution were determined by nitrogen adsorption–desorption measurements using the Brunauer–Emmett–Teller (BET) method (Micromeritics Gemini IV). The pore size distribution was further analyzed using the Barrett–Joyner–Halenda (BJH) model to evaluate mesoporous characteristics.

### 2.7 Electrochemical characterization

All electrochemical measurements were carried out at room temperature using a Gamry reference 3000 electrochemical workstation. For the three-electrode configuration, the prepared Ag–MnO_2_/MXene electrode was used as the working electrode, Ag/AgCl (3 M KCl) as the reference electrode, and platinum foil as the counter electrode in 2 M KOH aqueous electrolyte. Cyclic voltammetry (CV) measurements were performed within a potential window of 0 to 0.6 V at various scan rates ranging from 5 to 100 mV s^−1^. Galvanostatic charge–discharge (GCD) tests were conducted at different current densities (1–20 A g^−1^) to evaluate specific capacitance and rate capability. Electrochemical impedance spectroscopy (EIS) measurements were performed over a frequency range from 100 kHz to 0.01 Hz with an amplitude of 5 mV to analyze charge transfer resistance and ion diffusion behavior.

The working electrode was prepared by mixing; 85 wt% active material, 10 wt% acetylene black, 5 wt% PVDF. The slurry was dispersed in NMP and coated onto pre-treated nickel foam (1 × 1 cm^2^). The electrodes were dried at 80 °C for 12 h and pressed under 10 MPa to improve contact. Mass loading was controlled at ∼1–1.2 mg cm^−2^. For symmetric supercapacitor (SSC) fabrication, identical Ag–MnO_2_/MXene electrodes were used as both positive and negative electrodes. In the case of asymmetric supercapacitor (ASC), Ag–MnO_2_/MXene was used as the positive electrode, while MXene served as the negative electrode. A filter paper separator was immersed in 2 M KOH electrolyte until full saturation. The separator was then sandwiched between two electrodes and pressed gently to ensure proper contact. The assembled device was stabilized at room temperature for 15 min prior to measurements.

The electrochemical performance of the assembled devices was evaluated *via* CV, GCD, and EIS measurements under similar conditions as the three-electrode system. The operating voltage window of the device was optimized based on stability tests.

Eqn (1) was applied to calculate the specific capacitance (*S*_c_) of the electrodes at different scan rates. In this equation, *S*_c_ (F g^−1^) denotes the specific capacitance, ∫*I*(v)d*V* represents the integrated area under the current–potential curve, *v* (mV s^−1^) is the scan rate, *m* (g) corresponds to the mass of the active material, and Δ*V* (V) indicates the potential window.1
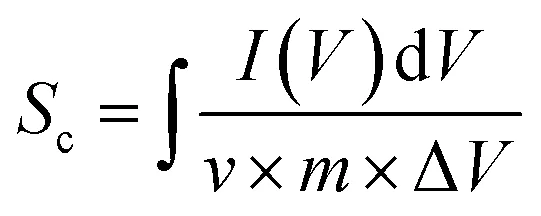


Eqn (2) and (3) were employed to determine the specific capacitance (*S*_c_) of the electrodes based on galvanostatic charge–discharge (GCD) profiles. These equations describe the relationship between the discharge current (*I*, A), discharge time (*t*, s), applied potential window (*V*, V), and the geometric area of the electrode (cm^2^). The current density (*J*, A g^−1^) was calculated by normalizing the applied current with respect to the mass of the active material (*I*/*m*).2
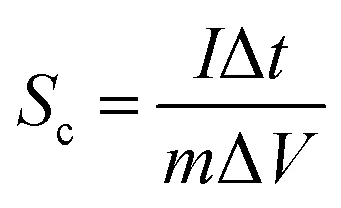
3
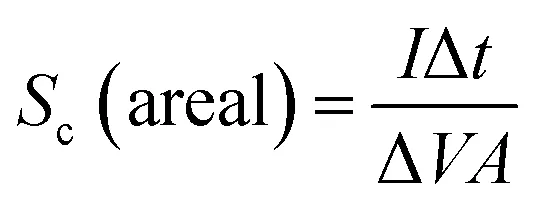


To evaluate measurement reproducibility, specific capacitance measurements for the Ag–MnO_2_/MXene electrode were performed on three independently fabricated electrodes (*n* = 3); results are reported as mean ± standard deviation. Other electrochemical parameters (MnO_2_ and MnO_2_/MXene reference electrodes, cycling stability) were obtained from single representative measurements.

### 2.8 Advanced electrochemical kinetic analysis

To further elucidate the charge storage behavior of the Ag–MnO_2_/MXene hybrid electrode, a comprehensive kinetic analysis was conducted based on cyclic voltammetry (CV) and electrochemical impedance spectroscopy (EIS) data.

#### 2.8.1 *b*-Value determination

The relationship between peak current and scan rate was analyzed by plotting log(*i*) *versus* log(*v*). The slope of the fitted linear curve was used to determine the *b*-value, which provides insight into the dominant charge storage mechanism. A *b*-value approaching 1 indicates a surface-controlled capacitive process, whereas a value close to 0.5 suggests diffusion-limited behavior.^39^

#### 2.8.2 Capacitive contribution quantification

The total current response at a fixed potential was separated into capacitive and diffusion-controlled contributions. The capacitive contribution ratio was calculated at different scan rates to evaluate the dominance of surface-controlled processes. The variation of capacitive contribution with increasing scan rate was systematically analyzed to reveal the kinetic advantages of the hybrid structure.

#### 2.8.3 Diffusion behavior and ion transport analysis

The diffusion-controlled contribution was further examined to understand ion transport within the porous electrode structure. The presence of MXene layers and interconnected MnO_2_ nanosheets was expected to facilitate ion diffusion by providing shortened diffusion pathways and enhanced electrolyte accessibility.

#### 2.8.4 Electrochemical impedance analysis and equivalent circuit modeling

Electrochemical impedance spectroscopy (EIS) data were analyzed using an equivalent circuit model to extract key kinetic parameters, including solution resistance (*R*_s_), charge transfer resistance (*R*_ct_), and Warburg impedance (*Z*_w_).

The Nyquist plots were interpreted to evaluate the effect of biogenic Ag nanoparticles on charge transfer kinetics. A reduced semicircle diameter in the high-frequency region indicates lower *R*_ct_, while the slope in the low-frequency region reflects improved ion diffusion.

#### 2.8.5 Synergistic effect evaluation

To quantitatively assess the synergistic contribution of each component, comparative analyses were performed among MnO_2_, MXene, MnO_2_/MXene, and Ag–MnO_2_/MXene systems.

The enhancement in electrochemical performance was attributed to: the pseudocapacitive redox activity of MnO_2_, the high electrical conductivity and layered structure of MXene and the improved interfacial charge transfer induced by biogenic Ag nanoparticles.

#### 2.8.6 Rate capability and kinetic stability

The rate capability was evaluated by analyzing capacitance retention at increasing current densities. The improved retention at high scan rates and current densities was correlated with the dominant capacitive contribution and reduced diffusion resistance.

## 3 Results and discussion

### 3.1 Characterization results

Fig. 2(a) presents the XRD patterns of MnO_2_, MnO_2_/MXene, and Ag-decorated MnO_2_/MXene composites. The pristine MnO_2_ exhibits diffraction peaks at 2*θ* ≈ 12.7°, 18.1°, 28.8°, 37.4°, 49.8°, and 60.2°, corresponding to the (110), (200), (310), (211), (411), and (521) planes, respectively, consistent with the tetragonal, tunnel-structured α-MnO_2_ phase (JCPDS card no. 44-0141).^4^ The tunnel-type crystal structure of α-MnO_2_, formed by double chains of MnO_6_ octahedra, provides one-dimensional (2 × 2) channels that facilitate cation (K^+^/H^+^) intercalation and are expected to contribute favorably to the pseudocapacitive charge storage behavior observed in this study. After hybridization with MXene, no additional impurity peaks are observed, while a slight change in peak intensity suggests strong interfacial interaction between the α-MnO_2_ phase and the conductive MXene layers. In the Ag-integrated sample, additional diffraction reflections indexed to metallic Ag (JCPDS no. 04-0783; (111), (200), and (220) planes at 2*θ* ≈ 38.1°, 44.3°, and 64.4°, respectively) confirm the successful incorporation of biogenic silver nanoparticles. The absence of unwanted secondary phases supports the phase purity of the synthesized hybrid. The preserved α-MnO_2_ reflections together with the Ag peaks indicate that the tunnel-structured phase is retained after composite formation. The pristine MnO_2_ exhibits characteristic diffraction peaks corresponding to its crystalline phase, indicating successful formation of the oxide structure. After hybridization with MXene, no additional impurity peaks are observed, while a slight change in peak intensity suggests strong interfacial interaction between MnO_2_ and the conductive MXene layers. In the Ag-integrated sample, additional diffraction reflections indexed to metallic Ag (*e.g.*, (111), (200), and (220)) confirm the successful incorporation of biogenic silver nanoparticles. The absence of unwanted secondary phases supports the phase purity of the synthesized hybrid.^40–42^ The preserved MnO_2_ reflections together with Ag peaks indicate that the structural integrity of MnO_2_ is maintained after composite formation.

**Fig. 2:**
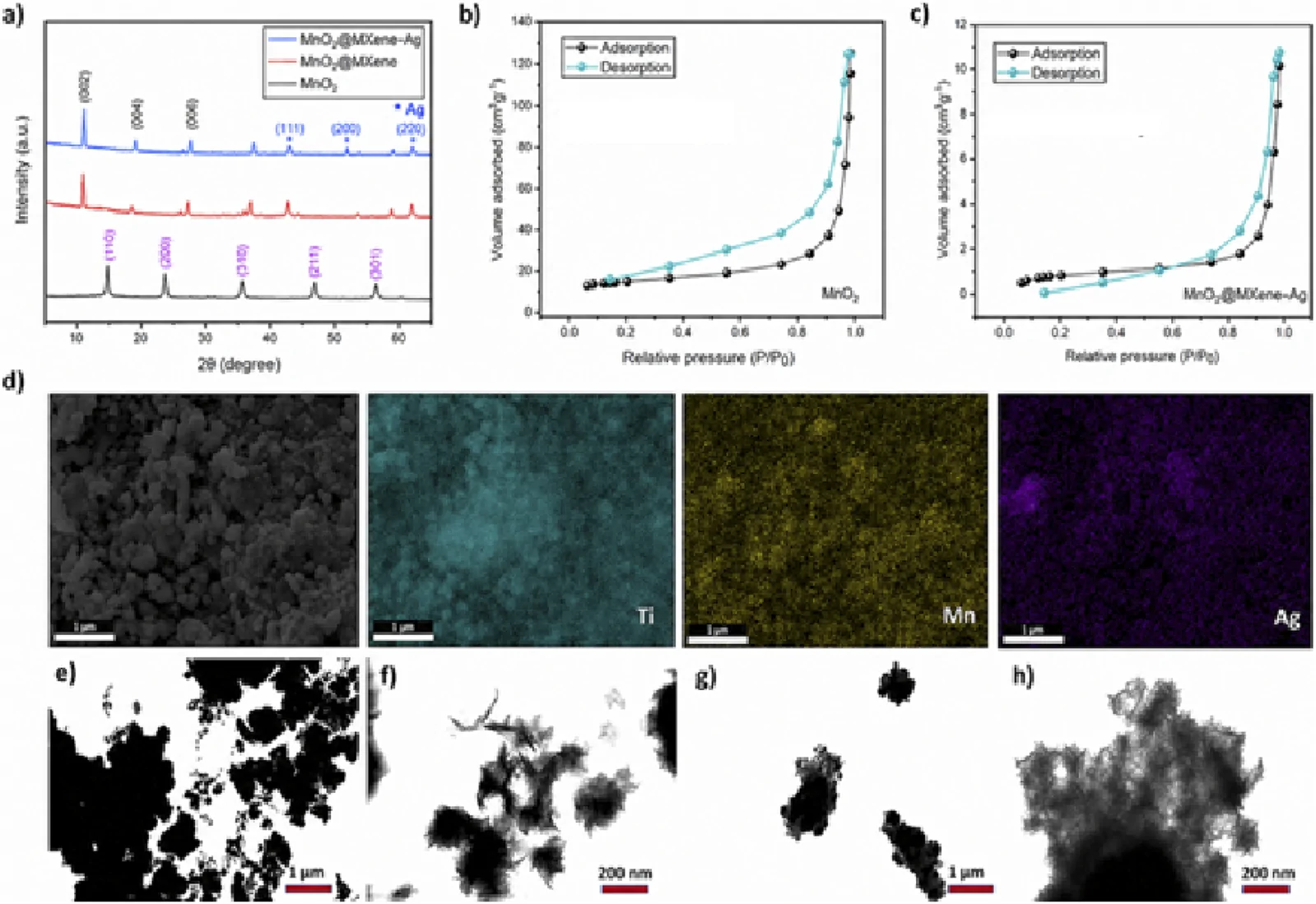
(a) X-ray diffraction (XRD) patterns of the materials. Nitrogen adsorption–desorption isotherm of the (b) MnO_2_ and (c) MnO_2_@MXene. (d) Elemental mapping of the materials. STEM image of the (e) MnO_2_, (f) MnO_2_@MXene, (g) and (h) MnO_2_@MXene-Ag.

Fig. 2(b) and (c) show the N_2_ adsorption–desorption isotherms of MnO_2_ and Ag–MnO_2_/MXene, respectively. Both samples display type-IV isotherms with a distinct hysteresis loop, characteristic of mesoporous materials.^43^

The specific surface area of pristine MnO_2_ is calculated as 31 m^2^ g^−1^. After integration with MXene and Ag nanoparticles, the surface area decreases to 2.72 m^2^ g^−1^. This reduction can be attributed to the partial occupation of pores and surface sites by MXene sheets and Ag nanoparticles. The specific surface area of pristine MnO_2_ is calculated as 31 m^2^ g^−1^, whereas that of the Ag–MnO_2_/MXene composite decreases markedly to 2.72 m^2^ g^−1^. Although MXene incorporation is often expected to preserve or enhance accessible surface area, this reduction is consistent with the well-known restacking behavior of two-dimensional MXene nanosheets, wherein van der Waals interactions and hydrogen bonding between surface terminal groups (–OH, –O, –F) promote re-aggregation of the delaminated flakes during composite formation, substantially reducing the interlayer spacing and accessible surface area.^31^ This effect is further compounded by partial occlusion of MXene interlayer galleries and MnO_2_ pore structures by the anchored Ag nanoparticles and MnO_2_ nanosheets.

Despite this reduction in BET surface area, the retained type-IV isotherm and hysteresis loop confirm that the mesoporous character of the composite is preserved, providing continuous ion-diffusion pathways. Taken together with the substantially reduced charge-transfer resistance observed by EIS (Fig. 4(a–c)) and the enhanced surface-controlled capacitive contribution indicated by the Dunn's-method analysis, these results suggest that electrochemical performance in this system is governed primarily by interfacial charge-transfer kinetics and electrical conductivity rather than by total BET surface area alone consistent with previous reports on MXene-based composites in which restacking-induced surface area loss does not preclude enhanced capacitive performance.^44^ Electrochemical impedance spectroscopy (EIS) measurements were fitted to an equivalent circuit model to extract the solution resistance (*R*_s_), charge-transfer resistance (*R*_ct_), and Warburg impedance (*W*) for each electrode. The Ag–MnO_2_/MXene electrode exhibited the lowest solution resistance (*R*_s_ = 0.4995 Ω) among the three systems, compared to 0.5839 Ω for MnO_2_ and 0.6979 Ω for MnO_2_/MXene, indicating improved electrolyte accessibility and electrical contact upon Ag and MXene integration. The charge-transfer resistance followed the order MnO_2_ (*R*_ct_ = 2.40 × 10^−4^ Ω) < Ag–MnO_2_/MXene (*R*_ct_ = 4.33 × 10^−4^ Ω) < MnO_2_/MXene (*R*_ct_ = 5.44 × 10^−4^ Ω), while the Warburg impedance was highest for MnO_2_ (2.53 × 10^−2^ Ω), intermediate for the Ag-integrated ternary electrode (2.19 × 10^−2^ Ω), and lowest for MnO_2_/MXene (1.54 × 10^−2^ Ω), reflecting differences in ion-diffusion pathways across the three architectures.

Despite the decrease in BET surface area, the composite structure provides improved electrical pathways and electrochemically active interfaces, which are more critical for charge storage than surface area alone. The presence of mesoporosity facilitates ion diffusion within the electrode.

Fig. 2(d) shows the SEM image along with elemental mapping results. The MnO_2_ exhibits a clustered and interconnected morphology, forming a porous network. Such a structure is beneficial for electrolyte penetration. Elemental mapping confirms the uniform distribution of Ti (from MXene), Mn and O (from MnO_2_), and Ag across the composite. The C signal is uniformly distributed and consistent with the underlying MXene carbon framework, while the O signal reflects the combined contribution of the MnO_2_ oxide lattice and the oxygen-containing surface terminations (–O, –OH, –F) characteristic of MXene, collectively confirming successful integration of the oxide and MXene phases.

Elemental mapping confirms the uniform distribution of Ti (from MXene), Mn, and Ag throughout the composite. The homogeneous dispersion of Ag nanoparticles indicates that the biogenic synthesis route enables effective anchoring without severe agglomeration. This uniformity supports efficient electron transport and active site accessibility.^43^

Fig. 2(e–h) present STEM images at different magnifications. The MnO_2_ shows aggregated nanostructures with irregular morphology, while the composite exhibits a more open and interconnected architecture due to the presence of MXene sheets.^44^

At higher magnification, thin and layered MXene structures decorated with MnO_2_ and Ag nanoparticles are clearly observed. The Ag nanoparticles appear as darker contrast regions, indicating their successful deposition on the MnO_2_/MXene framework.

The nanoscale integration between MnO_2_, MXene, and Ag provides shortened ion diffusion paths and enhanced electrical conductivity. This hierarchical structure supports fast charge transfer and contributes to improved electrochemical performance.

The biogenic synthesis route may also influence the interfacial characteristics of the Ag nanoparticles. During the reduction of Ag^+^ by *Camellia japonica* stamen extract, plant-derived compounds can act as reducing and stabilizing species and may remain partially associated with the AgNP surface after isolation and washing. Such surface-associated organic components may contribute to nanoparticle stabilization and dispersion and may also influence the interaction of AgNPs with the MnO_2_/MXene framework. However, the present characterization does not allow individual phytochemical species to be assigned unambiguously; therefore, their contribution is considered a possible interfacial effect rather than a confirmed chemical mechanism. The electrochemical enhancement observed after Ag incorporation is consequently discussed primarily in terms of the conductive and interfacial role of Ag, while acknowledging that residual plant-derived surface species may additionally influence the local electrode environment.

The CV curves of the MnO_2_ electrode Fig. 3(a) exhibit relatively low enclosed area with distinguishable redox peaks, indicating limited capacitive contribution. In the case of the MnO_2_/MXene electrode Fig. 3(b), a noticeable increase in the integrated area and peak current is observed, suggesting improved charge transport facilitated by the conductive MXene network. The Ag–MnO_2_/MXene electrode Fig. 3(c) delivers the highest current response and the largest CV area among all samples. The sharper and more symmetric redox peaks indicate enhanced reversibility and faster redox kinetics. This behavior is attributed to the presence of Ag nanoparticles, which act as conductive bridges, promoting electron transfer and increasing the electrochemically active surface.^45–47^

The MnO_2_ electrode Fig. 3(d) shows a relatively short discharge time along with a pronounced IR drop, reflecting limited capacitance and higher internal resistance. The MnO_2_/MXene electrode Fig. 3(e) demonstrates an extended discharge duration and a reduced IR drop, indicating improved conductivity and ion/electron transport. The Ag–MnO_2_/MXene electrode Fig. 3(f) exhibits the longest discharge time and the smallest IR drop. The nearly symmetric charge–discharge profiles confirm high coulombic efficiency and stable electrochemical behavior. These results confirm that Ag incorporation significantly enhances charge storage capability and electrode kinetics.^48^

**Fig. 3:**
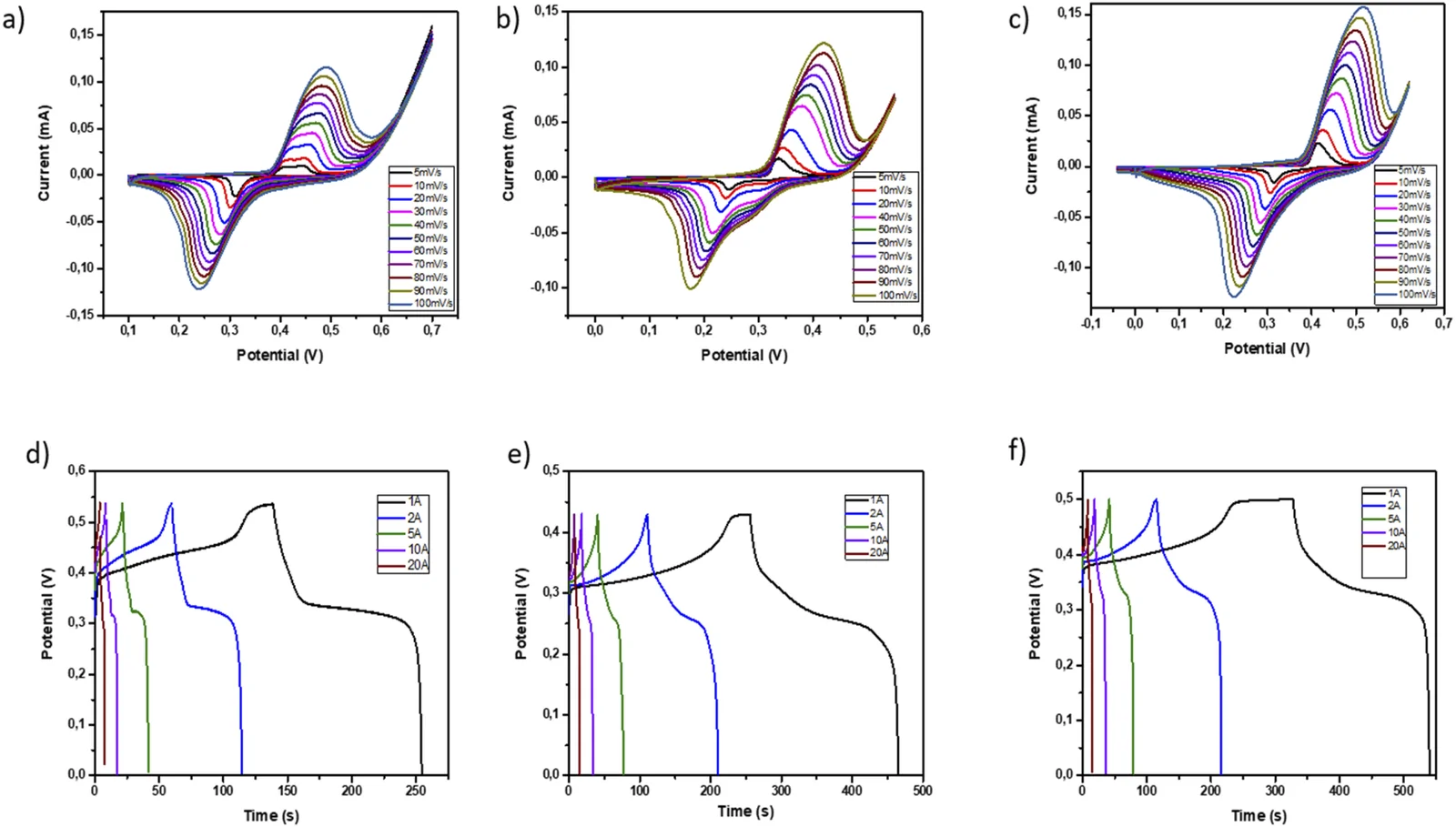
Electrochemical performance of the prepared electrodes: (a) CV curves of the MnO_2_ electrode, (b) CV curves of the MnO_2_/MXene electrode, (c) CV curves of the Ag–MnO_2_/MXene electrode, (d) galvanostatic charge–discharge curves of the MnO_2_ electrode, (e) galvanostatic charge–discharge curves of the MnO_2_/MXene electrode, (f) galvanostatic charge–discharge curves of the Ag–MnO_2_/MXene electrode.

**Fig. 4:**
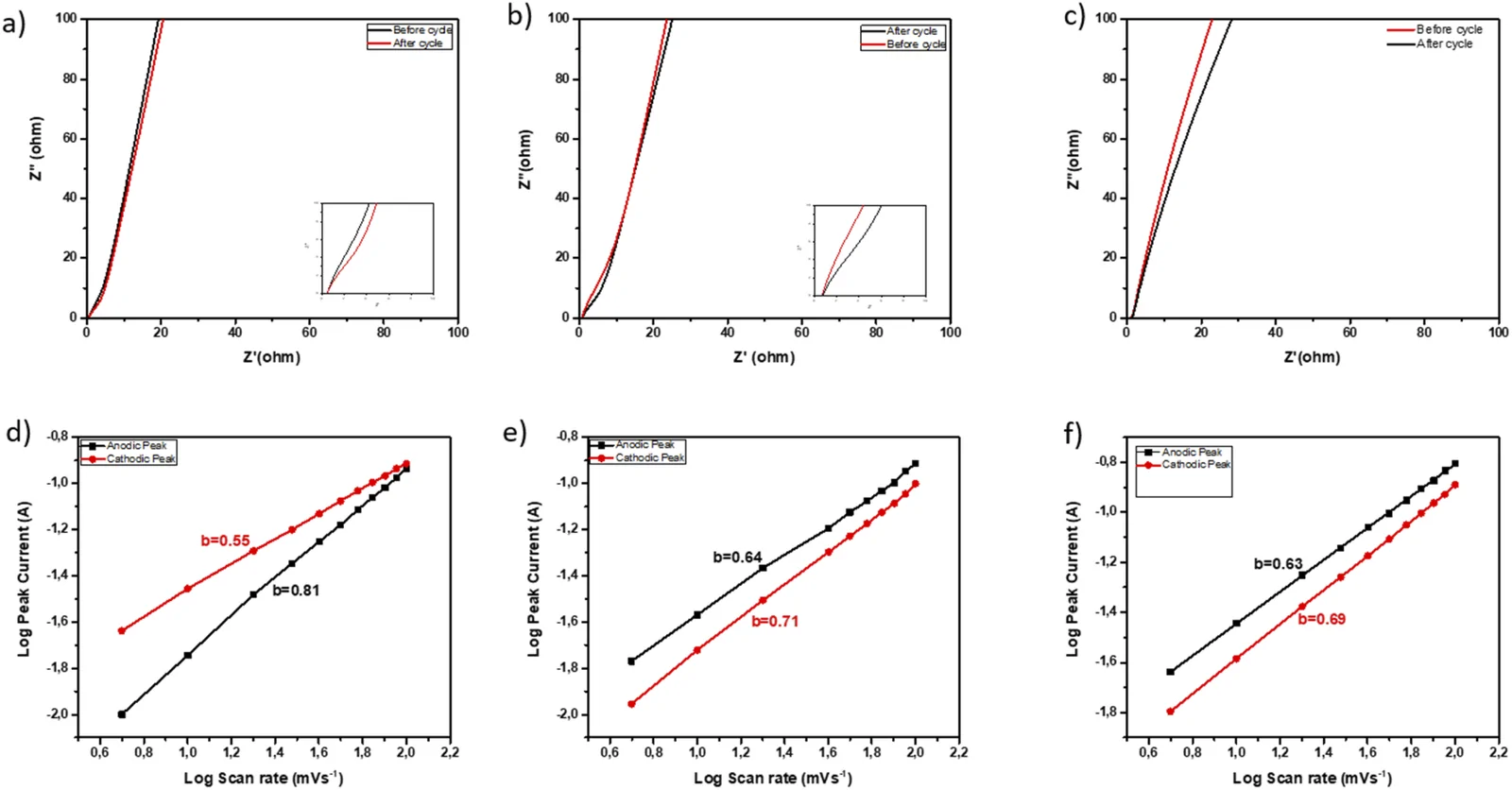
(a) Nyquist impedance plots of the MnO_2_ electrode before and after cycling, (b) Nyquist impedance plots of the MnO_2_/MXene electrode before and after cycling, and (c) Nyquist impedance plots of the Ag–MnO_2_/MXene electrode before and after cycling (d–f) the logarithm of peak currents *vs.* the logarithm of scan rates.

The Nyquist plot of the MnO_2_ electrode Fig. 4(a) presents a large semicircle in the high-frequency region, corresponding to high charge-transfer resistance (*R*_ct_), along with a less steep slope in the low-frequency region, indicating hindered ion diffusion. For the MnO_2_/MXene electrode Fig. 4(b), the semicircle diameter decreases and the low-frequency line becomes steeper, confirming improved conductivity due to the MXene framework. The Ag–MnO_2_/MXene electrode Fig. 4(c) displays the smallest semicircle and a nearly vertical line in the low-frequency region, approaching ideal capacitive behavior. The retention of this feature after cycling demonstrates low *R*_ct_ and strong electrochemical stability of the hybrid structure.^49–51^

A comparative assessment of all electrochemical results indicates that the Ag–MnO_2_/MXene nanohybrid electrode delivers the highest performance in terms of current response, discharge duration, and charge-transfer characteristics. This enhancement arises from the synergistic interaction between the conductive MXene network and the electron-bridging role of Ag nanoparticles. The Ag–MnO_2_/MXene electrode delivers a specific capacitance of 782.6 ± 8.8 F g^−1^ at 1 A g^−1^ (*n* = 3), representing a substantial improvement over the MnO_2_ and MnO_2_/MXene reference electrodes.

The MnO_2_/MXene electrode Fig. 4(e) exhibits a further increase in *b*-value relative to pristine MnO_2_, consistent with a greater contribution from surface-controlled capacitive behavior due to improved electrical conductivity and more accessible active sites. Interestingly, the Ag-integrated ternary electrode (Fig. 4(f)) displays a comparable, marginally lower *b*-value (0.63/0.69) relative to the binary MnO_2_/MXene system, despite its superior overall electrochemical performance (Fig. 3–5). The *b*-values, extracted from the slopes of the log(*i*) − log(*v*) plots for the anodic and cathodic peaks, were 0.55/0.81, 0.64/0.71, and 0.63/0.69 for the MnO_2_, MnO_2_/MXene, and Ag–MnO_2_/MXene electrodes, respectively. This apparent discrepancy suggests that Ag nanoparticle incorporation introduces additional faradaic (diffusion-influenced) redox contributions alongside the enhanced conductivity provided by the MXene network, rather than shifting the mechanism further toward a purely surface-controlled regime. Consequently, the *b*-value alone does not fully capture electrode performance; the superior current response, discharge duration, and reduced charge-transfer resistance of the Ag–MnO_2_/MXene electrode instead point to a synergistic combination of fast electron transport and enhanced redox activity, further supported by the Dunn's-method capacitive/diffusive contribution analysis.^52^

**Fig. 5:**
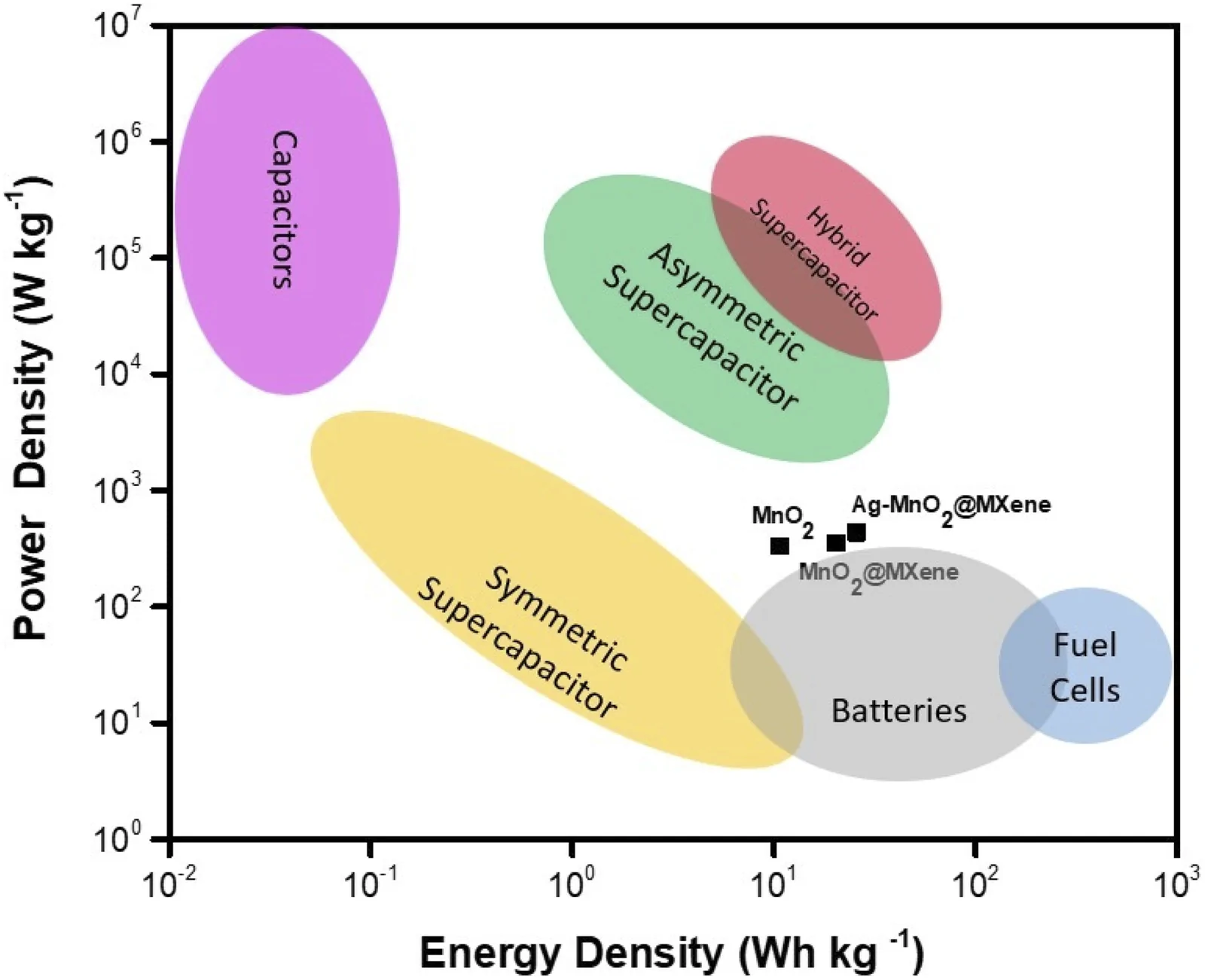
Ragone plots showing the relationship between energy density and power density for MnO_2_, MnO_2_/MXene, and Ag–MnO_2_/MXene electrodes, highlighting the superior energy–power performance of the Ag-integrated hybrid system.

The galvanostatic charge–discharge curves recorded at different current densities further confirm this trend. The MnO_2_ electrode displays a rapid decrease in discharge time as the current density increases, indicating limited rate capability and higher internal resistance. In comparison, the MnO_2_/MXene electrode maintains longer discharge durations with reduced voltage drop, demonstrating improved charge transport. The Ag–MnO_2_/MXene electrode delivers the longest discharge times across all applied current densities, with well-preserved curve symmetry.^53–55^ This behavior indicates superior rate performance, lower internal resistance, and enhanced electrochemical stability. The improved performance is associated with the conductive MXene framework and the presence of Ag nanoparticles, which facilitate faster electron transport and accelerate redox reactions within the electrode structure.^56^

Fig. 5 Ragone plot showing the relationship between energy density and power density for MnO_2_, MnO_2_/MXene, and Ag–MnO_2_/MXene electrodes. The Ag-integrated hybrid electrode exhibits a superior balance of high energy and high power density compared to pristine MnO_2_ and MnO_2_/MXene, approaching the ideal region between conventional capacitors and batteries, highlighting its potential for advanced energy storage applications.

To contextualize the electrochemical performance of the Ag–MnO_2_/MXene electrode developed in this study, Table 1 benchmarks its synthesis method, electrolyte, current density, specific capacitance, energy/power density, and cycling stability against recently reported (2021–2026) MXene/MnO_2_-based and Ag-decorated ternary supercapacitor systems. The specific capacitance obtained here (782.6 ± 8.8 F g^−1^ at 1 A g^−1^) is competitive with comparable ternary systems in the literature; however, unlike the majority of benchmarked systems, long-term cycling stability was not evaluated in the present study and is identified as an important direction for future work.

**Table 1:** Comparison of the electrochemical performance of the Ag–MnO_2_/MXene electrode developed in this study with recently reported (2021–2026) MXene/MnO_2_-based and Ag-decorated ternary supercapacitor electrodes

System	Synthesis method	Electrolyte	Current density	Capacitance	Energy/power density	Cycling stability	Ref.
Ag–MnO_2_/MXene (this work)	Electrostatic adsorption (plant-mediated AgNPs + MILD-etched MXene + MnO_2_ deposition)	KOH (aq.)	1 A g^−1^	782.6 ± 8.8 F g^−1^	∼22–25 Wh kg^−1^/∼400–450 W kg^−1^ (approx., read from Ragone plot)	Not evaluated in this study	—
Ag–MnO_2_/MXene@NF	Hydrothermal + post-sonication, Ag-doping	Not specified	1 A g^−1^	1188 F g^−1^	—	96.4% (6000 cycles)	57
MXene/MnO_2_/Ag (2D/1D/0D)	Integration of 2D MXene, 1D tubular MnO_2_, 0D Ag nanospheres	Not specified	2 A g^−1^	880.8 F g^−1^	39.9 Wh kg^−1^@2955.8 W kg^−1^ (ASC)	85.0% (5000 cycles)	58
MXene@WS_2_/MnO_2_	Hierarchical hydrothermal synthesis	Not specified	5 mV s^−1^ (scan rate)	824.7 F g^−1^	30.6 Wh kg^−1^@440.7 W kg^−1^ (ASC)	87.6% (5000 cycles)	59
MnO_2_@MXene@CC	Sequential immersion (8 cycles)	0.5 M Na_2_SO_4_	1 A g^−1^	234.8 F g^−1^	9.6 Wh kg^−1^@902.3 W kg^−1^ (ASC)	Not specified	60
MnO_2_@MXene (ultrasonic)	Ultrasonication	3 M H_2_SO_4_	1.5 A g^−1^	133.28 F g^−1^	101.71 Wh kg^−1^@2670 W kg^−1^	Not specified	61

## 4 Conclusions

In this study, MnO_2_, MnO_2_/MXene, and Ag–MnO_2_/MXene electrodes were successfully synthesized and thoroughly characterized. XRD, BET, SEM/STEM, and elemental mapping analyses confirmed the structural integrity, mesoporosity, and homogeneous distribution of MXene and Ag nanoparticles within the hybrid framework. Electrochemical evaluations, including cyclic voltammetry, galvanostatic charge–discharge, Nyquist impedance, and Ragone plots, demonstrated that the Ag–MnO_2_/MXene hybrid electrode exhibits superior performance compared to pristine MnO_2_ and MnO_2_/MXene. Specifically, the Ag-integrated system achieves enhanced charge-transfer kinetics, higher current response, longer discharge times, reduced internal resistance, and a favorable balance of energy and power density. These improvements are attributed to the synergistic effects of MXene's conductive network and the electron-bridging role of Ag nanoparticles, which facilitate fast ion diffusion and efficient electron transport. Overall, the Ag–MnO_2_/MXene hybrid represents a promising candidate for high-performance energy storage applications, combining both high power and energy densities while maintaining excellent electrochemical stability.

## Conflicts of interest

The authors declare that they have no known competing financial interests or personal relationships that could have appeared to influence the work reported in this manuscript.

## Data Availability

The data supporting the findings of this study are available from the corresponding author upon reasonable request.
